# Effects of Rapid Maxillary Expansion on Upper Airway Volume in Growing Children: A Three-Dimensional Cone-Beam Computed Tomography Study

**DOI:** 10.7759/cureus.34274

**Published:** 2023-01-27

**Authors:** Mohammed A Korayem

**Affiliations:** 1 Orthodontics Department of Preventive Dental Sciences, Albaha University College of Dentistry, Albaha, SAU

**Keywords:** orthodontic apparatus, volume of upper portion of airway, rapid maxillary expansion, retrospective analysis, cbct

## Abstract

*Background: *Rapid maxillary expansion (RME) is a common orthodontic procedure that widens the maxillary arch to treat moderate to mild overcrowding and transverse skeletal and dental abnormalities. Orthodontic equipment applies lateral tension on posterior maxilla teeth or palate mucosa to the mid-palatal suture. The maxilla may grow transversely when force is applied at right angles to the mid-palatal suture, which is usually inactive in children and adolescents. This study used cone-beam computed tomography (CBCT) and an authorized upper respiratory airway volume measurement approach to compare RME cohort pharyngeal airway volume changes to healthy controls.

*Materials and Methods: *This retrospective analysis included 52 RME patients and 52 healthy controls. The RME category's expansion regimen entailed twisting the screw of expansion on a tooth-attached Hyrax-type expansion equipment by 0.25 mm daily for at least 14 days. After six months, a few RME participants used fixed orthodontic gear. The comparison group used fixed orthodontic appliances for minor malocclusions without extractions (without RME). CBCT scans from 1021 orthodontic patients who visited a dental hospital between 2012 and 2022 were examined. The registry comprised only anonymized photographs. Volume, minimum cross-sectional area (MCA), molar width, and inter-molar width were measured before and after therapy.

*Results: *The control group had 12227.12 mm^3^ at T0 and 15805.54 mm^3^ at T1. The control group's T0-T1 volume difference was statistically significant (p = 0.007). The RME group has 12884.84 mm^3^ at T0 and 17471.08 mm^3^ at T1. The RME group had a significant volume difference at T0 and T1 (p = 0.002). The volume RME effect was ±1011.92 and statistically insignificant. (p > 0.05). MCA in the control group was 126.04 mm^2^ at T0 and 170.61 mm^2 ^at T1. MCA at T0 and T1 in the control group was statistically significant (p = 0.041). RME group MCA was 126.53 mm^3^ at T0 and 164.69 mm^2^ at T1. The RME group had a significant volume difference at T0 and T1 (p = 0.002). The MCA, RME effect was 5.92 and statistically insignificant (p > 0.05). Both the control and RME groups had statistically significant volume and MCA differences at T0 and T1. However, the intergroup analysis showed no significant differences across the groups.

*Conclusion:* Tooth-borne RME does not affect upper airway or MCA volume in children compared to controls. Upper airway changes were better with younger skeletal ages before treatment. The findings may aid RME for young children.

## Introduction

In order to promote regular craniofacial maturation, early identification and intervention are preferred for the dentofacial abnormalities that appear as a consequence of upper airway compression [1]. The conditions of obstructive sleep apnea (OSA), sleep-disrupted breathing, and upper airway architecture have all been researched more frequently, and there is a common opinion that proper care at the beginning of such problems can have a better impact on patients' long-term general health outcomes as well as dental effects [2]. Rapid maxillary expansion (RME) is a frequent orthodontic procedure that increases the width of the maxillary arch to alleviate moderate to mild overcrowding of teeth while addressing transverse skeletal abnormalities and dental abnormalities [3]. Force is placed on the midpalatal suture by applying force in a lateral direction on the teeth present in the posterior region of the maxilla or mucosa of the palate using an orthodontic apparatus. When applied with force at right angles to the suture at the midpalatal region, which is typically a dormant condition in kids and adolescents, growth in the transverse direction in the maxilla may result [4-7]. RME's main goal is to apply force on the maxillary bone [8]. It has long been difficult to comprehend the three-dimensional impact of RME on the upper portion of the respiratory airway. Although auditory rhinometry has been employed, this method is only applicable to the nasal cavities [9-11]. The application of computed tomography has lately been discussed, although there are certain drawbacks, including the requirement that individuals be in a supine posture and a reasonably high dose of radiation [12,13]. It has been demonstrated that putting someone in certain positions alters the airway's volume [14-17].

Cone-beam computed tomography (CBCT) has been demonstrated to be a trustworthy and precise method for evaluating the upper portion of the respiratory airway in the upright position [18]. With easy-to-see markers and only a small amount of image magnification, it can see the edges between the airway spaces and the soft tissues next to them in both children and adults [19]. There are discrepancies in the data and a lack of uniformity across the measuring techniques utilized, according to a recent systematic assessment of earlier CBCT studies examining the variations in the upper portion of airways both before and after administration with RME [20].

The goals of this study were to compare changes in the volume of the pharyngeal airway as well as the minimum cross-sectional area (MCA) in an RME cohort with those in healthy controls using CBCT and an authenticated procedure for measuring the volume of the upper respiratory airway.

## Materials and methods

A registry of 1021 patients who visited a dental hospital for orthodontic therapy between 2012 and 2022 was evaluated to obtain their CBCT images. Ethical approval for the study was taken from the Albaha university with ref no: IEC/ALB/2022/11. All photos were anonymized before being included in the registry. The database also gave information about the person's gender, age, anatomical occlusion (based on Angle's classification of malocclusion), and the type of orthodontic therapy they were getting.

There were two categories in this retrospective analysis: a category of individuals with RME with 52 participants and a category of healthy controls with 52 participants. For a minimum period of 14 days, the expansion regimen in the category of RME involved turning the screw of expansion on a tooth-attached Hyrax-type appliance for expansion that was 0.25 mm each day. Few RME participants kept using fixed orthodontic appliances after the initial six-month duration of retention. In the comparison group, only fixed orthodontic appliances (no RME) were used to treat small misalignments without extractions.

The criteria for inclusion were: (1) RME procedure for unilateral or bilateral crossbite using a tooth-associated Hyrax expansion device; (2) a minimal rise of three mm in the distance between the molars on both sides of the maxilla was observed as a difference between the distance recorded in pre-treatment CBCT scans and the distance recorded in post-treatment CBCT scans, translating into a minimum anticipated orthopedic change of 1.5 mm in the upper jaw; (3) adolescents between the ages of 8 and 15; (4) cutting in regular intercuspal orientation with just an angled Class I molar contact; (5) Baseline CBCT scans and progression comprehensive scanning of the base of the cranium, maxillary bone, and mandibular bone, as well as the first six cervical vertebrae and related airways, is performed during CBCT scans.

Criteria for exclusion include prior history of orthodontic therapy, prior history of adenotonsillectomy, prior history of syndromic disorders, history of movement artifacts, prior history of swallowing while scanning and recording, and a treatment program necessitating the extraction of teeth for orthodontic treatment. After using criteria for who to include and who to leave out, the final cohort was made up of 52 patients.

Scan protocol: The same radiologist used the CBCT device of an iCAT Next Generation to capture the radiographic CBCT pictures. The settings were KVp of 120 Kv, current of 5 mA, voxel resolution of 0.4 mm, scan duration of 8.9 seconds, and a scanned space measuring 13 cm in height by 16 cm in diameter [21-23].

Image preparation and airway assessment (volume and MCA): The upper and lower margins, as per the previously established margins-which comprised the anterior margins and posterior boundaries of the airway-were specified in order to compute the MCA and volume. This was done to prevent the MCA from being calculated using a partial section caused by the discrepancy between the airway border for volume computation and the plane for computation. Within the set limits, the software automatically estimated the MCA (mm^2^) and volume (mm^3^). At the start of the active therapy (T0) and the completion of it, both measurements were performed (T1).

Assessment of the transverse effects of RME: For study participants at the start (T0) and end (T1) of treatment, posteroanterior-oriented CBCT-generated cephalometric radiographs were used to measure the width of the maxillary arch and the width of the mandible. In accordance with the procedure outlined by Yoon et al., these images were reconstructed by the digital software of CBCT with a minimum elongation of the radiographic image. To assess the skeletal treatment modifications attained with the RME apparatus, CBCT scans were obtained in a standard setting in RME study participants and then compared to images from the control group [23]. According to Adkins et al. [24], the intermolar dimension was determined using CBCT images taken from the extreme palatal side of the maxillary first molars at the position of the cementoenamel junction.

Statistical analysis: When put to the test using a Shapiro-Wilks assessment, the data sets from the RME category and control groups were both distributed evenly. A paired t-test was used to assess the dentofacial differences and airway distinctions among the two categories at T0. A multivariate regression model was used to analyze the intragroup and intergroup variations in the quantitative analysis of dentofacial structures and the upper portion of the respiratory airway at the beginning of treatment (T0) and the end of treatment (T1), taking into account the data's longitudinal and nested nature. The predictor variables, namely volume of the upper portion of the airway and MCA of the upper airway, and selected variables like the group of study participants and duration of the treatment procedure, in addition to their interactions, were all included in the fixed effects section of the models. The random effects part of the model was made up of the pairs of people who took part.

## Results

Volume in the control group at T0 was 12227.12 mm^3^, and at T1 was 15805.54 mm^3^. The difference in volume at T0 and T1 in the control group was quite relevant when statistically analyzed (p=0.007). Volume in the RME group at T0 was 12884.84 mm^3^, and at T1 was 17471.08 mm^3^. The difference in volume at T0 and T1 in the RME group was quite relevant when statistically analyzed (p=0.002). The RME effect regarding volume was ±1011.92, and it was not relevant when statistically analyzed (p > 0.05).

MCA in the control group at T0 was 126.04 mm^2^, and at T1 was 170.61 mm^2^. The difference in MCA at T0 and T1 in the control group was quite relevant when statistically analyzed (p=0.041). MCA in the RME group at T0 was 126.53 mm^3^, and at T1 was 164.69 mm^2^. The difference in volume at T0 and T1 in the RME group was quite relevant when statistically analyzed (p=0.002). The RME effect regarding MCA was 5.92, and it was not substantially important statistically (p > 0.05) (Table 1).

**Table 1:** Volume and MCA in control and RME group at T0 and T1 RME: Rapid maxillary expansion, MCA: Minimum cross-sectional area

	Volume (mm^3^)			MCA (mm^3^)		
	Control group	RME group	RME effect	Control group	RME group	RME effect
T0	12227.12	12884.84		126.04	126.53	
T1	15805.54	17471.08	±1011.92	170.61	164.69	5.92
P Value	0.007	0.002	0.67	0.041	0.031	0.101
95%Confidence Interval	13,258.24 to 18,330.62	15,028.06to 19,893.78	2401.10 to 4418.73	131.89 to 207.18	132.97 to 196.40	56.02 to 45.19

There were significant variations statistically in intra-group analysis in both the control group and RME group at T0 and T1 regarding volume and MCA. However, there were no substantially important distinctions between the two groups in the intergroup analysis.

Maxillary width in the control group at T0 was 59.67 mm, and at T1 was 61.28 mm. The difference in the width of the maxilla at the T0 stage and T1 stage in the control category was statistically significant (p=0.005). The maxillary width in the RME group at T0 was 59.91 mm, and at T1 was 62.81 mm. The difference in the width of the maxilla at the T0 stage and T1 stage in the RME category was statistically significant (p=0.002). The RME effect regarding maxillary width was -1.77, and it was not substantially important statistically (p > 0.05).

Maxillary inter-molar width in the control category at the T0 stage was 32.85 mm, and at the T1 stage was 33.73 mm. The difference in the width of the maxilla at the T0 stage and T1 stage in the control category was statistically significant (p=0.002). Maxillary inter-molar width in the RME category at the T0 stage was 31.42 mm, and at the T1 stage was 34.94 mm. The difference in maxillary width at T0 and T1 in the RME group was statistically significant (p=0.002). The RME effect regarding maxillary width was +3.83, and it was substantially important statistically (p=0.002) (Table 2).

**Table 2:** Maxillary width and maxillary intermolar width at T0 and T1 RME: Rapid maxillary expansion, MCA: Minimum cross-sectional area

	Maxillary width (mm)			Maxillary intermolar width (mm)		
	Control group	RME group	RME effect	Control group	RME group	RME effect
T0	59.67	59.91		32.85	31.42	
T1	61.28	62.81	-1.77	33.73	34.94	+3.83
P Value	0.005	0.002	0.006	0.002	0.002	0.002
95%Confidence Interval	59.42 to 62.14	61.95 to 63.68	0.51 to 2.91	33.39 to 34.86	33.26 to 35.74	2.44 to 4.42

There were significant variations statistically in intra-group analysis in both the control group and RME group at T0 and T1 regarding the width of the maxillary arch. Unluckily there were no substantially important distinctions between the two groups in the intergroup analysis. There were significant variations statistically in intra-group analysis in both the control group and RME group at T0 and T1 regarding maxillary inter-molar width (mm). There were no substantially important distinctions between the two groups on intergroup analysis.

## Discussion

The impacts of RME were examined by Cistulli et al. in a group of 10 individuals who had mild to moderate OSA [6]. All of these patients showed a decrease in the index score for distress in respiration; snoring was reduced in nine of these participants, along with a reduction in sleepiness during the daytime in these nine patients. In seven cases, the index for distress in respiration returned to normal. The authors concluded that RME might be a helpful therapeutic approach for some OSA patients [6]. Understanding the three-dimensional outcomes of RME on the upper portion of the respiratory airway has been a challenging task in the past. Although auditory rhinometry has been used, the nasal cavities are the only places where it may be used. The use of computed tomography has recently been addressed [7,8], although there are certain disadvantages, such as the need for subjects to be in the supine position and relatively large radiation dosage. Putting a person in certain positions has been shown to change the size of their airways [25-30]. Cistulli et al. investigated the effects of RME in a group of 10 people with mild to moderate OSA. Nine of these participants also had a decrease in snoring and daytime tiredness. All of these patients also showed a decrease in the respiratory distress score. Seven people had their respiration scores return to normal after experiencing respiratory distress. The authors concluded that, for some OSA patients, RME would be a beneficial therapy strategy [6].

The difference in volume at T0 and T1 in the control group was quite relevant when statistically analyzed (p = 0.007). The difference in volume at T0 and T1 in the RME group was quite relevant when statistically analyzed (p = 0.002). The RME effect regarding volume was 1011.92, and it was not relevant when statistically analyzed (p >0.05). MCA in the control group at T0 was 126.04 mm^2^, and at T1, it was 170.61 mm^2^. The difference in MCA at T0 and T1 in the control group was quite relevant when statistically analyzed (p = 0.041). MCA in the RME group at T0 was 126.53 mm^3^, and at T1, it was 164.69 mm^2^. The difference in volume at T0 and T1 in the RME group was quite relevant when statistically analyzed (p = 0.002). The RME effect regarding MCA was 5.92 and was not substantially important statistically (p >0.05).

There were significant variations statistically in intra-group analysis in both the control group and the RME group at T0 and T1 regarding volume and MCA. On intergroup analysis, however, there were no statistically significant differences between the two groups. Early detection and intervention are preferred for dentofacial abnormalities that develop as a result of upper airway compression to promote normal craniofacial maturation. Obstructive sleep apnea (OSA), sleep-disordered breathing, and upper airway architecture have all been studied more often, and it is generally believed that treating these conditions early may enhance patients' long-term dental and general health outcomes [31-35].

Rapid maxillary expansion (RME) is a common orthodontic procedure that widens the maxillary arch to treat transverse skeletal anomalies and dental irregularities, as well as moderate to mild tooth crowding. By exerting force in a lateral direction on the teeth located in the posterior part of the maxilla or the mucosa of the palate with the use of an orthodontic tool, force is applied to the mid-palatal suture. If pressure is put on the suture in the mid-palatal area, which is usually still present in children and teens, it can cause the maxilla to grow in a different direction [36-37]. The difference in maxillary width between the T0 and T1 stages in the control group was statistically significant (p = 0.005) in this study. In the RME category, the difference in maxillary width at T0 and T1 was statistically significant (p = 0.002). The RME effect regarding maxillary width was -1.77, and it was not substantially important statistically. (p > 0.05). Maxillary inter-molar width in the control category at the T0 stage was 32.85 mm, and at the T1 stage, it was 33.73 mm. In the control group, the difference in maxillary width at T0 and T1 was statistically significant (p = 0.002). It has been proven that cone-beam computed tomography (CBCT) is a reliable and accurate tool for assessing the upper section of the respiratory airway in the upright position. With easily identifiable markers and little image magnification, it can accurately identify the boundaries between the spaces of the airway and the surrounding soft tissues in both pediatric patients and adult patients.

In this study, maxillary inter-molar width in the RME category at the T0 stage was 31.42 mm, and at the T1 stage, it was 34.94 mm. The difference in maxillary width at T0 and T1 in the RME group was statistically significant (p = 0.002). The RME effect regarding maxillary width was 3.83, and it was substantially important statistically. (p=0.002). There were significant variations statistically in intra-group analysis in both the control group and the RME group at T0 and T1 regarding the width of the maxillary arch. Unfortunately, the intergroup analysis revealed no statistically significant differences between the two groups. There were significant variations statistically in the intra-group analysis in both the control group and the RME group at T0 and T1 regarding maxillary inter-molar width (mm). Based on the intergroup analysis, there were no statistically significant differences between the two groups. A review of past CBCT studies assessing the alterations in the upper region of the airways both before and after administration with RME revealed differences in the findings and a lack of homogeneity among the measuring methodologies used [38-44]. Anandarajah used CBCT in healthy, untreated individuals to determine a relationship between the volume of the upper section of the airway and the width of the maxillary and mandibular arches. Additionally, he recommended and supported a standardized method for measuring the upper airways [19]. The goal of this study was to measure the volume of the upper respiratory tract using a validated method, calculate the MCA, and compare the results to those of healthy controls using CBCT. Similar to what was found in other CBCT studies, there was no link between the position of the jaw and changes in the volume of the upper airway, or MCA.

## Conclusions

When compared to controls, tooth-borne RME is not linked to a substantial change in the volume of the upper airway or MCA in children. When the skeletal age prior to treatment was younger, the impact on the upper airway modifications was more favorable. The findings could be useful, particularly in the RME procedure for young kids.

## References

[1] McNamara JA (1981). Influence of respiratory pattern on craniofacial growth. Angle Orthod.

[2] Magliocca KR, Helman JI (2005). Obstructive sleep apnea: diagnosis, medical management and dental implications. J Am Dent Assoc.

[3] McNamara JA Jr (2002). Early intervention in the transverse dimension: is it worth the effort?. Am J Orthod Dentofacial Orthop.

[4] Işeri H, Tekkaya AE, Oztan O, Bilgiç S (1998). Biomechanical effects of rapid maxillary expansion on the craniofacial skeleton, studied by the finite element method. Eur J Orthod.

[5] Bishara SE, Staley RN (1987). Maxillary expansion: clinical implications. Am J Orthod Dentofacial Orthop.

[6] Cistulli PA, Palmisano RG, Poole MD (1998). Treatment of obstructive sleep apnea syndrome by rapid maxillary expansion. Sleep.

[7] Doruk C, Sökücü O, Sezer H, Canbay EI (2004). Evaluation of nasal airway resistance during rapid maxillary expansion using acoustic rhinometry. Eur J Orthod.

[8] Ceroni Compadretti G, Tasca I, Alessandri-Bonetti G, Peri S, D'Addario A (2006). Acoustic rhinometric measurements in children undergoing rapid maxillary expansion. Int J Pediatr Otorhinolaryngol.

[9] Doruk C, Sökücü O, Biçakçi AA, Yilmaz U, Taş F (2007). Comparison of nasal volume changes during rapid maxillary expansion using acoustic rhinometry and computed tomography. Eur J Orthod.

[10] Garib DG, Henriques JFC, Janson G, Freitas MR, Coelho RA (2005). Rapid maxillary expansion—tooth tissue-borne versus tooth-borne expanders: a computed tomography evaluation of dentoskeletal effects. Angle Orthod..

[11] Martin SE, Mathur R, Marshall I, Douglas NJ (1997). The effect of age, sex, obesity and posture on upper airway size. Eur Respir J.

[12] Neill AM, Angus SM, Sajkov D, McEvoy RD (1997). Effects of sleep posture on upper airway stability in patients with obstructive sleep apnea. Am J Respir Crit Care Med.

[13] Yildirim N, Fitzpatrick MF, Whyte KF, Jalleh R, Wightman AJ, Douglas NJ (1991). The effect of posture on upper airway dimensions in normal subjects and in patients with the sleep apnea/hypopnea syndrome. Am Rev Respir Dis.

[14] Jan MA, Marshall I, Douglas NJ (1994). Effect of posture on upper airway dimensions in normal human. Am J Respir Crit Care Med.

[15] Guijarro-Martínez R, Swennen GR (2011). Cone-beam computerized tomography imaging and analysis of the upper airway: a systematic review of the literature. Int J Oral Maxillofac Surg.

[16] Weissheimer A, Menezes LM, Sameshima GT, Enciso R, Pham J, Grauer D (2012). Imaging software accuracy for 3-dimensional analysis of the upper airway. Am J Orthod Dentofacial Orthop.

[17] Di Carlo G, Saccucci M, Ierardo G (2017). Rapid maxillary expansion and upper airway morphology: A systematic review on the role of cone beam computed tomography. Biomed Res Int.

[18] Anandarajah S, Abdalla Y, Dudhia R, Sonnesen L (2015). Proposal of new upper airway margins in children assessed by CBCT. Dentomaxillofac Radiol.

[19] Anandarajah S, Dudhia R, Sandham A, Sonnesen L (2017). Risk factors for small pharyngeal airway dimensions in preorthodontic children: A three-dimensional study. Angle Orthod.

[20] Garrett BJ, Caruso JM, Rungcharassaeng K, Farrage JR, Kim JS, Taylor GD (2008). Skeletal effects to the maxilla after rapid maxillary expansion assessed with cone-beam computed tomography. Am J Orthod Dentofacial Orthop.

[21] Baccetti T, Franchi L, McNamara JA, Jr Jr (2002). An improved version of the cervical vertebral maturation (CVM) method for the assessment of mandibular growth. Angle Orthod.

[22] Bjork A (1947). The face in profile: an anthropological X-ray investigation on Swedish children and conscripts. Sven Tandlak Tidskr.

[23] Yoon YJ, Perkiomaki MR, Tallents RH (2004). Transverse craniofacial features and their genetic predisposition in families with nonsyndromic unilateral cleft lip and palate. Cleft Palate Craniofac J.

[24] Adkins MD, Nanda RS, Currier GF (1990). Arch perimeter changes on rapid palatal expansion. Am J Orthod Dentofacial Orthop.

[25] (1940). Statistical Methods for Medical and Biological Students. Br Med J.

[26] Houston WJB (1983). The analysis of errors in orthodontic measurements. Am J Orthod.

[27] Solow B, Siersbaek-Nielsen S, Greve E (1984). Airway adequacy, head posture, and craniofacial morphology. Am J Orthod.

[28] Muto T, Yamazaki A, Takeda S (2006). Relationship between the pharyngeal airway space and craniofacial morphology, taking into account head posture. Int J Oral Maxillofac Surg.

[29] Zhao Y, Nguyen M, Gohl E, Mah JK, Sameshima G, Enciso R (2010). Oropharyngeal airway changes after rapid palatal expansion evaluated with cone-beam computed tomography. Am J Orthod Dentofacial Orthop.

[30] El H, Palomo JM (2014). Three-dimensional evaluation of upper airway following rapid maxillary expansion: a CBCT study. Angle Orthod.

[31] Baratieri CdL, Alves M, Jr Jr, Mattos CT, Lau GWT, Nojima LI, de Souza MMG (2014). Transverse effects on the nasomaxillary complex one year after rapid maxillary expansion as the only intervention: a controlled study. Dent Press J Orthod..

[32] Chang Y, Koenig LJ, Pruszynski JE, Bradley TG, Bosio JA, Liu D (2013). Dimensional changes of upper airway after rapid maxillary expansion: a prospective cone-beam computed tomography study. Am J Orthod Dentofacial Orthop.

[33] Sadeghian S, Ghafari R, Feizbakhsh M, Dadgar S (2016). Dimensional changes of upper airway after rapid maxillary expansion evaluated with cone beam computed tomography. Orthod Waves.

[34] Schendel SA, Jacobson R, Khalessi S (2012). Airway growth and development: a computerized 3-dimensional analysis. J Oral Maxillofac Surg.

[35] Zeng J, Gao X (2013). A prospective CBCT study of upper airway changes after rapid maxillary expansion. Int J Pediatr Otorhinolaryngol.

[36] Pangrazio-Kulbersh V, Wine P, Haughey M, Pajtas B, Kaczynski R (2012). Cone beam computed tomography evaluation of changes in the naso-maxillary complex associated with two types of maxillary expanders. Angle Orthod.

[37] Iwasaki T, Takemoto Y, Inada E (2014). Three-dimensional cone-beam computed tomography analysis of enlargement of the pharyngeal airway by the Herbst appliance. Am J Orthod Dentofacial Orthop.

[38] Kim SY, Park YC, Lee KJ (2018). Assessment of changes in the nasal airway after nonsurgical miniscrew-assisted rapid maxillary expansion in young adults. Angle Orthod.

[39] Cantarella D, Dominguez-Mompell R, Moschik C (2018). Midfacial changes in the coronal plane induced by microimplant-supported skeletal expander, studied with cone-beam computed tomography images. Am J Orthod Dentofacial Orthop.

[40] Sonnesen L, Petersson A, Berg S, Svanholt P (2017). Pharyngeal airway dimensions and head posture in obstructive sleep apnea patients with and without morphological deviations in the upper cervical spine. J Oral Maxillofac Res.

[41] Rabah N, Al-Ibrahim HM, Hajeer MY, Ajaj MA (2022). Evaluation of rapid versus slow maxillary expansion in early adolescent patients with skeletal maxillary constriction using cone-beam computed tomography: A short-term follow-up randomized controlled trial. Dent Med Probl.

[42] Al-Ouf K, Krenkel C, Hajeer MY, Sakka S (2010). Osteogenic uni- or bilateral form of the guided rapid maxillary expansion. J Craniomaxillofac Surg.

[43] Alsawaf DH, Almaasarani SG, Hajeer MY, Rajeh N, Rajeh N (2022). The effectiveness of the early orthodontic correction of functional unilateral posterior crossbite in the mixed dentition period: a systematic review and meta-analysis. Prog Orthod.

[44] Husson AH, Burhan AS, Hajeer MY, Nawaya FR (2021). Three-dimensional oropharyngeal airway changes after facemask therapy using low-dose computed tomography: a clinical trial with a retrospectively collected control group. Prog Orthod.

